# Utility of transversus abdominis plane block on patients undergoing transanal total mesorectal excision

**DOI:** 10.12669/pjms.39.2.7062

**Published:** 2023

**Authors:** Jun Ying, Chunhui Jiang, Chunjie Xu, Ye Liu, Lei Gu

**Affiliations:** 1 Jun Ying, Department of Anesthesiology, Renji Hospital, School of Medicine Shanghai Jiao Tong University, Shanghai 200127, P.R. China; 2 Chunhui Jiang, Department of Gastrointestinal Surgery, Renji Hospital, School of Medicine Shanghai Jiao Tong University, Shanghai 200127, P.R. China; 3 Chunjie Xu, Department of Gastrointestinal Surgery, Renji Hospital, School of Medicine Shanghai Jiao Tong University, Shanghai 200127, P.R. China; 4 Ye Liu, Department of Gastrointestinal Surgery, Renji Hospital, School of Medicine Shanghai Jiao Tong University, Shanghai 200127, P.R. China; 5 Lei Gu, Department of Gastrointestinal Surgery, Renji Hospital, School of Medicine Shanghai Jiao Tong University, Shanghai 200127, P.R. China

**Keywords:** Transversus abdominis plane block, Anesthesia, Rectal cancer, Transanal total mesorectal excision, Natural orifice specimen extraction surgery

## Abstract

**Objective:**

To evaluate the analgesic effect of transversus abdominis plane block (TAPB) on patients undergoing transanal total mesorectal excision (taTME).

**Methods:**

Medical records of patients who were eligible to receive proctectomy surgery in Renji Hospital, Shanghai Jiao Tong University School of Medicine (From January 2019 to December 2021) were retrospectively reviewed. Propensity score matching (PSM) was applied to the included cases. A total of 120 cases were divided into three groups based on the different operation and anesthesia methods used. Group-A (Lap, n=40) included patients that underwent laparoscopic surgery under general anesthesia. Group- B (ta, n=40) included patients who received taTME surgery under general anesthesia. Group-C (ta+TAPB, n=40) included patients who received taTME surgery under general anesthesia combined with TAPB. The dosage of sufentanil, time of postoperative revival and extubation, anal exhaust time and other adverse events were recorded. Pain assessment using the visual analogue scale (VAS) was performed at 12, 24,48 and 72 hours after the operation.

**Results:**

There were no significant differences in the general parameters, operative conditions, and anesthetic administration between the three groups (P>0.05). The dosage of sufentanil was significantly reduced in Group-C, compared with Group-A and Group-B, with no difference between the groups A and B. There was no significant difference between the three groups in postoperative recovery time and extubation time. VAS score was lower in Group-C than Group-A and Group-B. This difference was more obvious in the early postoperative period and gradually diminished with time. VAS score became similar in all three groups 72 hours after the surgery.

**Conclusion:**

Transanal total mesorectal excision was associated with less pain, compared to laparoscopic TME. TAPB with general anesthesia in patients undergoing taTME is safe and effective. It can significantly reduce the use of sufentanil and has optimal analgesic effect.

***List of abbreviations:***

**taTME:** Transanal total mesorectal excision; **TAPB:** Transversus abdominis plane block;

**PCIA:** Patient controlled intravenous analgesia; **VAS:** Visual analogue scale;

**NOSES:** Natural orifice specimen extraction surgery; **ERAS:** Enhanced recovery after surgery.

## INTRODUCTION

Total mesorectal excision (TME) is considered the gold standard treatment for colorectal cancer (CRC).^1^ Transanal TME (TaTME) combines abdominal and transanal endoscopic approaches, allows to reduce the abdominal incision and improves the postoperative abdominal pain.^2^ Transversus abdominis plane (TAP) block is the injection of local anesthetics to neurofascial plane between internal oblique and transversus abdominis muscles and is highly effective in reducing perioperative pain.^3^,^4^ The purpose of this study was to explore whether TAPB is beneficial to the recovery of patients undergoing taTME procedure.

## METHODS

We performed a retrospective analysis of CRC patients who were eligible to receive proctectomy surgery in Renji Hospital, Shanghai Jiao Tong University School of Medicine from January 2019 to December 2021.

### Inclusion Criteria:

- No distant metastasis;
- No obstruction;
- No emergency surgery;
- No radiotherapy or chemotherapy and other anti-tumor treatment;
- No history of other malignant tumors;
- No colorectal multiple primary cancer.

### Exclusion criteria:

- Distant metastasis;
- Large bowl obstruction;
- Emergency surgery;
- Radiotherapy or chemotherapy and other anti-tumor treatment;
- History of other malignant tumors;
- Multiple primary colorectal cancer.

Propensity score matching (PSM) was used to select inclusive cases of each group. Gender(M/F), Age (years), BMI (kg/m2), The American Society of Anesthesiologists (ASA) score (I/II/III) and surgery duration(min) were variables that influenced the results of the study and were used as control variables to match CRC cases and to screen out comparable samples in the three groups. A total of 120 cases were divided into three groups according to the different operation and anesthesia methods used. Group-A (Lap, n=40) contained patients that underwent laparoscopic surgery under general anesthesia, Group-B (ta, n=40) included patients that underwent taTME surgery under general anesthesia, and Group-C (ta+TAPB, n=40) included patients that received taTME surgery under general anesthesia combined with TAPB.

### Ethics approval and Consent to participate:

All patients provided written informed consent before the operation, and this study was approved by Ethics Committee of Renji Hospital, School of Medicine Shanghai Jiao Tong University (Number of ethics approval: KY2019-014) and carried out in accordance with the ethical standards formulated in the Helsinki Declaration.

The rectum was mobilized according to TME guidelines for both laparoscopy and transanal procedures. The specimen was removed from lower midline incision by the laparoscopic surgery after being pulled out through anus by the taTME surgery (Fig.1). All patients were given sodium lactate ringer injection through venous access. Blood pressure, heart rate, SpO2 and etCO2 were monitored. Anesthesia was induced and included midazolam 0.04mg/kg, etomidate 0.3mg/kg, sufentanil 3-4μg/kg, rocuronium 0.6mg/kg. After tracheal intubation, mechanical ventilation was performed. Propofol 4-8mg/(kg·h), remifentanil 0.05-0.10μg/(kg·h) and rocuronium 0.15mg/(kg·h) were continuously pumped during the operation. No intraoperative analgesia was administered as per guidelines of our hospital. Sufentanil-based patient controlled intravenous analgesia (PCIA) was used in all groups with the same regimen for 48 hours after the surgery.

**Fig.1:**
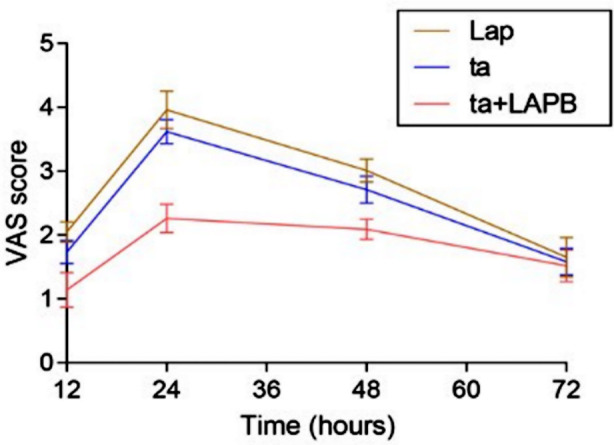
VAS after surgery

**Fig.1a:**
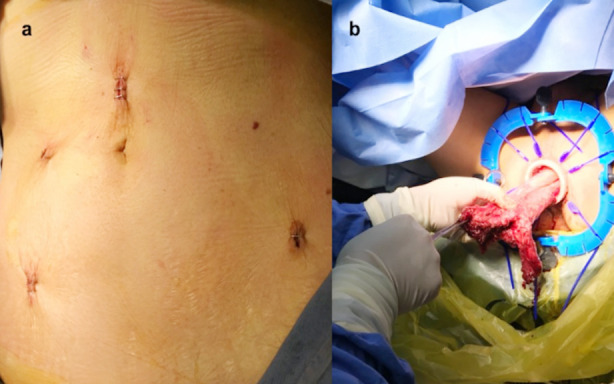
The specimen was removed after being pulled out through anus. a) 4 trocar sites in the abdomen; b) rectum removed from anus

**Fig.2:**
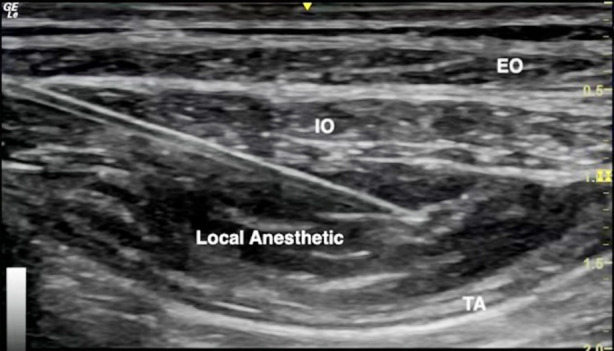
Ultrasound guided TAPB (EO: external oblique, IO: internal oblique, TA: transversus abdominis)

### Ultrasound guided TAPB:

TAPB was performed immediately by a qualified anesthesiologist using ultrasound guidance (GE LOGIQ E) and a broadband (4 to 13 MHz) linear array ultrasound probe. The probe was placed transversely in the midaxillary line between the iliac crest and the costal margin.^5^ A 22G 100-mm nerve block needle was inserted when the transversus abdominis plane (TAP) was identified. When the tip of the needle was in the TAP, bilateral block was performed with the injection of 25mL of 0.25% ropivacaine (Fig.2). The dosage of sufentanil, time of postoperative revival and extubation, anal exhaust time and other adverse events were recorded. Pain assessment using the visual analogue scale (VAS) was performed at 12,24,48 and 72 hours after the operation. Data collection was done by physicians, namely, the first two authors (JY, of the Department of Anaesthesia, and CJ of the Department of Gastroenterology).

### Statistical analysis:

All categorical data were counted as cases or percentages, and continuous data were expressed as mean ± SD. Statistical analyses were conducted by Statistical Product and Service Solutions (SPSS) 20.0 and Graph Pad Prism-five software. Categorical data were analyzed using the Chi-squared (χ^2^) test or Fisher’s exact test. Multivariate analysis was performed through Multivariate Cox proportional hazards regression analysis. P<0.05 was considered statistically significant.

## RESULTS

The perioperative characteristics of the three groups are shown in Table-I. There were no significant differences in general parameters, operation conditions and anesthetic administration among three groups (P>0.05). The dosage of sufentanil was significantly reduced in Group-C, compared with Groups A and B. There was no difference in the dosage of sufentanil between Groups A and B. There was no significant difference among three groups in postoperative recovery time and extubation time (Table-II). No other forms of analgesia were used during the study time.

**Table-I:** Perioperative characteristics of the three groups.

	Group-A (Lap, n=40)	Group-B (ta, n=40)	Group-C (ta+TAPB, n=40)	P value
Gender (M/F)	25/15	26/14	22/18	0.635
Age (years)	59.48±7.6	60.22±8.0	61.05±7.8	0.906
BMI (kg/m2)	24.56±3.2	23.71±3.4	24.02±2.8	0.475
ASA (I/II/III)	1/26/13	1/28/11	1/31/8	0.798
Surgery duration (min)	170.23±42.63	181.26±40.01	175.76±38.62	0.604
Blood loss (ml)	71.23±20.85	63.15±18.62	67.68±17.83	0.171
Hospital stay (day)	8.16±0.68	8.01±0.62	8.25±0.52	0.211

**Table-II:** Comparison of sufentanil dosage and postoperative recovery.

	Group-A (Lap, n=40)	Group-B (ta, n=40)	Group-C (ta+TAPB, n=40)	P value
Dosage of sufentanil (μg,x ± s)	24.60±1.81	25.34±1.09	18.76±0.96	<0.0001
Time of postoperative revival (min,x ± s)	13.24±1.28	13.62±1.71	12.91±0.81	0.059
Time of extubation (min,x ± s)	16.21±2.21	16.28±1.92	16.51±1.73	0.775
Exhaust time (min,x ± s)	92.31±4.52	93.17±4.16	91.66±4.82	0.327
Urinary retention (n, %)	12.50%	10%	7.50%	0.755
Nausea and vomiting (n, %)	25%	30%	22.50%	0.739

VAS score was lower in Group-C compared to Group-A and Group-B. The difference in the VAS score was more obvious in the early postoperative period and diminished gradually with the increase in the postoperative time. Seventy-two hours after the surgery, there was no difference in the VAS scores between three groups (Table-III) and (Fig.1a).

**Table-III:** VAS after surgery

VAS	Group-A (Lap, n=40)	Group-B (ta, n=40)	Group-C (ta+TAPB, n=40)	P value
12h after surgery	2.05±0.16	1.73±0.18	1.14±0.27	<0.0001
24h after surgery	3.96±0.29	3.62±0.19	2.26±0.22	<0.0001
48h after surgery	3.01±0.18	2.71±0.21	2.09±0.16	<0.0001
72h after surgery	1.65±0.31	1.58±0.21	1.52±0.25	0.086

## DISCUSSION

Our study shows that the level of pain in patients after taTME is lower compared to laparoscopic TME. We demonstrated that TAPB with general anesthesia is effective method in patients undergoing taTME and is not associated with the increased risk of adverse effects.

Laparoscopy has become a routine procedure in colorectal surgery, especially for low rectal cancer.^6^,^7^ It has an irreplaceable advantage in the exposure of some surgical fields. The development of pneumoperitoneum may have a certain influence on anesthesia, and the rise of diaphragm affects the effective ventilation of lung. Good muscle relaxation and adequate sedation, as well as good postoperative analgesia, can help patients recover better.

There is still no consensus on whether the specimen extraction site matters in rectal surgery. Although it is not mandatory, natural orifice specimen extraction surgery (NOSES) is often used in taTME surgery. In the current study, all cases of taTME had no abdominal incision, and the specimens were pulled out through the anus. Previous reports suggested that the type of abdominal incision does not affect the required postoperative analgesic dosage.^8^ As NOSES requires no additional incisions for the extraction of the lesion, it is causing less physical trauma^9^,^10^ and is associated with better postoperative outcomes, lower pain scores and lesser need for analgesics.^11^,^12^ Our study showed that although there was no difference in the dosage of sufentanil, the VAS score was slightly improved in taTME group in our study, suggesting that the specimen extraction site has a certain effect on the postoperative analgesia.

TAPB was first described in 2001 and has since undergone multiple modifications.^13^,^14^ The ultrasound (US)-guided TAPB was first described in 2007.^15^-^17^ The aim in all cases of TAM is to block some or all of the lower six thoracic spinal nerves (T7-T12) and the iliohypogastric and ilioinguinal nerves (L1). A recent meta-analysis concluded that TAPB confers a statistically significant analgesic benefit in adult patients undergoing abdominal laparotomy, laparoscopy, or cesarean delivery.^18^ The routine use of TAPB in cesarean section further proves its safety.^19^-^21^ In recent years, studies have found that TAPB can not only play an analgesic effect, but also alleviate visceral pain.^22^ At the same time, TAPB can effectively block the conduction of peripheral surgical noxious stimulation to reduce patients’ perception of pain and help to prevent the occurrence of hyperalgesia. Our study also confirmed that patients with TAPB can get better experience and analgesic effect.

TAPB is often mentioned in the context of enhanced recovery after surgery (ERAS).^23^-^25^ ERAS was first described in 1997 and widely used in gastrointestinal surgery now.^26^-^28^ It is a clinical practice process that integrates evidence-based medicine in perioperative period, makes anesthesia, surgery and nursing team cooperate effectively, adopts the best clinical path, reduces trauma stress, promotes early recovery of organs and psychology in perioperative period to reduce perioperative complications and shorten length of hospital stay. However, in the operation of rectal cancer, especially low rectal cancer, the implementation of ERAS must be performed with caution^29^ because of the risk of potential anastomotic leakage and intra-abdominal infection.^30^ In agreement with these observations, our study also did not detect significant differences in the length of hospital stay among the three groups

The postoperative pain in patients with rectal cancer mainly comes from the incision of abdominal wall and the contraction of visceral smooth muscle. Even with the same stimulation, everyone has different tolerance for pain. The nature of pain makes objective measurement impossible.^31^ The perception of pain varies greatly among individuals, and the cognition of colleagues on the nature of pain varies greatly with their own experience and language expression. The currently accepted methods of pain assessment include visual analogue scale (VAS), numerical rating scale (NRS) or verbal rating scale (VRS).^32^,^33^ As suggested by numerous studies, there is still a need to develop multi-dimensional assessment instruments.^34^ We selected VAS for pain assessment as it can be more objective to make a score in the same assessment system, and is simple and sensitive. A better scale can be used in future studies to assess the degree of pain.

Up to date, there are only few reports on postoperative analgesia in patients that undergo taTME. The results of our study may, therefore, provide the starting-point data of the patient’s experience of post-taTME analgesia to illustrate the importance of the quality of life and subjective experience of such patients. Our results may influence the overall decision making process of clinicians, selecting the best approach for the colorectal surgery.

### Limitations:

It is a single-center retrospective analysis. Further prospective multi-center studies with larger sample sized are needed.

## CONCLUSION

The level of pain in patients after taTME is lower compared to laparoscopic TME. TAPB with general anesthesia on patients undergoing taTME is safe and effective. Our results have clear clinical implications as they allow clinicians to choose surgical approach that significantly reduce the use of sufentanil and has an ideal analgesic effect.

### Availability of data and materials:

The datasets used and analyzed during the current study are available from the corresponding author on reasonable request.

### Authors’ contributions:

**JY and CJ** conceived and designed the study.

**CX, YL and LG** collected the data and performed the analysis.

**JY and CJ** were involved in the writing of the manuscript and are responsible for the integrity of the study.

All authors have read and approved the final manuscript.
